# Vitamin abnormalities in neuropathic corneal pain

**DOI:** 10.1186/s40662-025-00457-x

**Published:** 2025-10-12

**Authors:** Bing Jie Chow, Mingyi Yu, Chang Liu, Isabelle Xin Yu Lee, Louis Tong, Yu-Chi Liu

**Affiliations:** 1 Barts and the London School of Medicine and Dentistry, Queen Mary University of London, London, UK; 2 Tissue Engineering and Cell Therapy Group, Singapore Eye Research Institute, The Academia, 20 College Road, Level 6, Singapore, 169856 Singapore; 3 Ocular Surface Research Group, Singapore Eye Research Institute, Singapore, Singapore; 4 Department of Cornea and External Eye Disease, Singapore National Eye Centre, Singapore, Singapore; 5 Ophthalmology and Visual Sciences Academic Clinical Program, Duke-NUS Medical School, Singapore, Singapore

**Keywords:** Vitamins, Corneas, Neuropathic corneal pain, Ocular pain

## Abstract

Neuropathic corneal pain (NCP) refers to spontaneous corneal pain in the absence of stimuli arising from corneal nerve dysfunction with no clinically observable ocular surface abnormalities. It is debilitating with difficult-to-manage symptoms—burning pain, photophobia, and irritation being profound. However, evidence-based clinical recommendations for the management of NCP remain scarce. Given the established role of vitamins in various neuropathies and associations between vitamin deficiencies and NCP in the literature, vitamin supplementation represents a potential therapeutic avenue that has yet to be adequately investigated in the context of NCP. This narrative review provides an overview of the therapeutic potential of vitamins B3, B12 and D as treatment in NCP, drawing evidence from both preclinical animal and clinical studies. It discusses the potential mechanisms of action rendered by various vitamins in alleviating NCP and includes the suppression of inflammation, neuroinflammation, mitochondrial dysfunction, oxidative stress, as well as the modulation of neurodegeneration and nociception dysregulation. Furthermore, we offer insight on future directions needed for vitamin supplementation to serve as mainstream treatment for NCP. Future research should also aim to establish optimal treatment protocols, including dosing regimens, treatment duration and administration methods for each vitamin.

## Background

Neuropathic corneal pain (NCP) is a complex and ill-defined disease entity characterised by aberrant pain perception in the absence of painful stimuli. The hallmark features of NCP include burning, sensitivity to wind and light, electric-shock-like, or stabbing pain, as well as corneal hyperalgesia and allodynia, often without detectable commensurate clinical signs [1]. These symptoms are typically refractory to standard dry eye therapies. Given the cornea’s role as the most richly innervated tissue in the body [2], the condition can be profoundly debilitating, markedly diminishing quality of life alongside significant psychological and economic repercussions [3, 4]. NCP has been reported to occur postoperatively in refractive surgery patients, chronic dry eye disease (DED) and contact lens users, while also occurring in diabetes mellitus, neurotrophic keratopathy, systemic chronic pain syndromes and autoimmune conditions [5, 6].

**Fig. 1:**
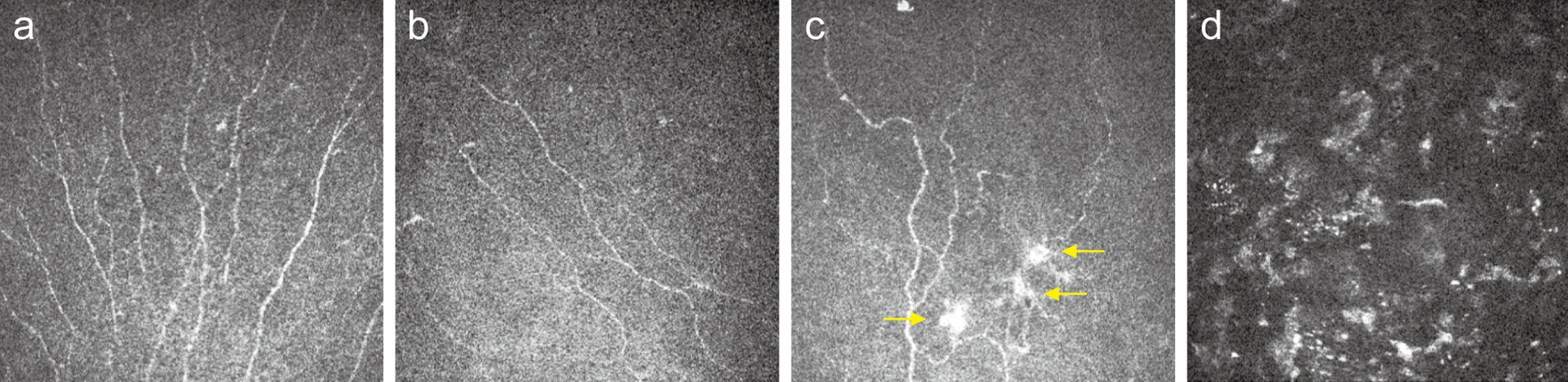
Representative in vivo confocal microscopy (IVCM) images from a healthy subject and a patient with neuropathic corneal pain (NCP). **a** Normal nerve morphology in a healthy subject. **b** Decreased corneal nerve density and corneal nerve fibre length in NCP. **c** Microneuromas (arrows) in NCP, manifested as irregularly shaped enlargements of terminal nerve endings with poorly defined margins and variable hyperreflectivity. **d** Activated keratocytes in NCP manifested as patchy areas of increased reflectivity in the stroma

Hypersensitization of peripheral or central corneal somatosensory nerves is thought to be the key driver, mediated by dual pathological mechanisms of inflammation and nociceptive signal amplification [7, 8]. While no definitive diagnostic tests exist for NCP, in vivo confocal microscopy (IVCM), which is classically used to visualise the corneal nerve plexus, has been reported to detect features indicative of NCP [7, 8]. These IVCM findings include the presence of microneuromas, significant decreases in corneal nerve fibre densities and length, as well as activated keratocytes (Fig. 1a–d) [1]. Measurement of tear molecular profile may also offer diagnostic value for NCP [9]. Additionally, validated instruments such as the Ocular Pain Assessment Survey (OPAS) and Ocular Surface Disease Index (OSDI) offer quantitative metrics for monitoring and comparing ocular pain symptoms and their associations [3, 10, 11]. Due to the diverse etiological spectrum, the characteristic clinical-symptomatic disparity, and the lack of standardised diagnostic and therapeutic frameworks, current insights into this condition remain fragmentary. Considering the current gaps in knowledge surrounding NCP, this review aims to outline current treatment strategies and explore the potential role and therapeutic efficacy of vitamin supplementation in mitigating associated symptoms of NCP.

## Main text

### Method of literature search

Electronic bibliographic searches in PubMed and Google Scholar from inception to 25 March 2025 were carried out for this narrative review with the primary research aim of examining the potential therapeutic efficacy of vitamin supplementation in NCP. The search strategy performed in the databases incorporated a range of keywords including “vitamin”, “vitamin deficiency”, “vitamin deficiencies”, “neuropathic corneal pain”, “neuropathic ocular pain”, “keratoneuralgia”, “neuropathic pain”, “corneal neuralgia”, “corneal”, “cornea”, “ocular surface”. Articles of all study types (clinical trials, meta-analyses, randomised controlled trials, review, and systematic review) pertaining to the application of vitamin supplementation in NCP patients were included in this article with duplicates eliminated. Articles not written in English or lacking full-text availability were excluded from the analysis. Relevant studies and publications identified through cross-referencing within the cited sources were also incorporated into the reference list to provide a more comprehensive discussion of the application of vitamin supplementation in NCP, and other neuropathic pain states, as well as the symptomatology, diagnosis, current treatment strategies and pathophysiology of NCP. A total of 65 articles were included in the final manuscript. No formal risk of bias assessment was conducted due to the narrative nature of the review.

### Current therapeutic approaches for NCP

The primary goals of therapeutic paradigms in NCP are the mitigation of inflammation and the promotion of corneal nerve regeneration [12]. Management of NCP frequently necessitates a multidisciplinary framework owing to its multisystem manifestations, involving synergistic efforts across ophthalmology, neurology, pain medicine, rheumatology, and psychiatry.

Given the pathogenic relationship between corneal nerve damage and neuroinflammation [13], anti-inflammatory therapy, including steroids and cyclosporine, constitutes a critical initial therapeutic approach for NCP [14]. Topical corticosteroids exert their anti-inflammatory effects through suppression of transcription factors responsible for pro-inflammatory cytokines activation, whereas cyclosporine exhibits immunomodulatory effects through suppression of lymphocytes and other pro-inflammatory cytokines within conjunctival tissues [15, 16].

In addition, emerging therapeutic approaches, such as blood-derived serum or plasma eye drops, have demonstrated efficacy in delivering essential growth factors such as vitamin A, epidermal growth factor, and fibronectin, while showing promising results in promoting neural regeneration and relieving pain symptoms [17, 18]. This is evidenced by significantly improved corneal nerve parameters and patient-reported symptom severity in NCP patients [17, 18]. Pharmacological interventions with oral neuromodulators such as anticonvulsants, serotonin-norepinephrine reuptake inhibitors (SNRIs) and tricyclic antidepressants have also been thought to offer therapeutic value for NCP relief, given their well-documented efficacy in treating generalised neuropathic pain [19, 20].

Nevertheless, the clinical management of NCP persists as a considerable clinical challenge, owing to the lack of a singular, reliably effective therapeutic intervention. Additionally, our current comprehension and clinical frameworks remain inadequate, with most therapeutic approaches to manage NCP derived from other fields of medicine. Given the established dual capacity of vitamins to provide neuroprotection and antioxidative effect, vitamin supplementations such as vitamins D and B have emerged for the treatment of neuropathic pain in a variety of neuropathic pain ranging from diabetic neuropathy, chemotherapy-induced neuropathic pain and fibromyalgia [21]. Additionally, current evidence suggest these supplements demonstrate synergistic effects when used in combination with existing pharmaceutical treatments for neuropathic pain [22]. Hence, vitamin supplementation presents as a promising therapeutic approach for NCP.

### Molecular pathogenesis of NCP

The underlying pathogenesis of NCP has been reported to be multifactorial, which encompasses several pathological processes ranging from neuroinflammation, mitochondrial dysfunction, apoptosis, neurotoxicity, wound healing, neuronal degeneration, to nociceptive dysfunction [1]. For example, metallothionein-2 was elevated in NCP [1], which is implicated in the initiation of inflammatory and neuropathic pain in response to noxious stimuli [23]. This is also reinforced by IVCM findings highlighting the presence of activated stromal keratocytes [1], where activation is commonly triggered by cytokines and growth factors in response to tissue injury caused by neuroinflammation [24]. The dysregulation in neurofilament light polypeptide, SNAP-25 and aquaporin-1 also highlights the role of nociceptive dysfunction in NCP, with these being established contributors to NCP and pain [25, 26]. Similarly, nerve degeneration in NCP was evidenced by the downregulation of bone morphogenetic protein 3 (BMP3) and dihydropyrimidinase-related protein 2 (DPYL2); BMP3 is known to facilitate peripheral nerve regeneration, while DPYL2 serves as a molecular marker for axonal growth and neural development [27]. Tear proteins implicated in mitochondrial dysfunction, such as nucleoside-diphosphate kinase 3 (NME3), have also been demonstrated to be dysregulated. Given the role of NME3 in mitochondrial fusion and motility, processes that influence synaptic energy homeostasis, mitochondrial dysfunction may play a role in NCP [5]. Mitochondrial dysfunction has also been implicated in the pathogenesis of peripheral neuropathic pain through upregulated translocator protein (TSPO) expression [28]. As a key modulator of mitophagy, TSPO dysregulation induces oxidative stress due to reactive oxygen species (ROS) accumulation and results in mitochondrial dysfunction [29]. These underlying molecular pathogenesis of NCP offers a glimpse of the interplay of the various pathological processes involved and may be targeted by therapy.

### Vitamin abnormalities and vitamin supplementation in NCP

#### Preclinical studies

Vitamin B3 has been investigated as a potential treatment for NCP; several preclinical studies suggested that neuropathic pain can be relieved by nicotinamide riboside, a member of the vitamin B3 family [30, 31]. Oral nicotinamide riboside supplementation at a dose of 3 g/kg of diet in prediabetic rats has been shown to reduce thermal hypoalgesia, restore impaired nerve conduction velocity, and prevent corneal nerve degeneration [32]. Similarly, in a separate rat model, 5-day treatment of nicotinamide riboside at a dose of 1000 mg/kg twice daily exhibited neuroprotective properties, attenuating neurite injury in spiral ganglia [33]. In an in vivo rat model by Hamity et al*.,* symptoms of NCP such as corneal hypersensitivity to tactile stimuli, were induced in the rats through intravenous administrations of paclitaxel for 5 days [34]. Compared to vehicle-treated rats, those administered paclitaxel showed markedly increased corneal sensitivity, with significantly lowered blink thresholds measured by Cochet-Bonnet aesthesiometry at 14 days after treatment. Nicotinamide riboside was subsequently administered at a dose of 500 mg/kg once daily for 28 days. Paclitaxel-induced corneal tactile hypersensitivity was significantly reversed in paclitaxel rats administered with nicotinamide riboside by the 11th day of administration, compared to paclitaxel rats treated with placebo. This suggests the potential therapeutic benefit of vitamin B3 in managing paclitaxel-associated neuropathic-like ocular pain, a potential finding of significant clinical relevance. At present, there are no published human studies documenting paclitaxel-induced NCP, although Chiang et al. reported that paclitaxel-treated group exhibited reduced corneal nerve fibre and inferior whorl lengths compared to controls [35]. Further research remains essential to better understand paclitaxel’s involvement in the context of ocular pain. Considering paclitaxel’s link with corneal nerve abnormalities [35], these findings suggest that nicotinamide riboside, an isoform of vitamin B3, may offer neuroprotective effects to alleviate symptoms of NCP such as corneal hypersensitivity.

#### Clinical studies

Clinical studies have found an association of vitamin abnormalities with NCP. One such study investigated a retrospective cohort of 84 patients with NCP [36]. Notably, vitamins B2 and D deficiencies were observed in 31% and 15.8% of cases, respectively, whereas elevated vitamins B6 and B12 levels were found in 28.9% and 15.0% of subjects. The reported elevation of vitamin B12 levels may appear contradictory to therapeutic supplementation in NCP. However, given the well-established neurotrophic properties of vitamin B12, we propose that its downregulation more likely reflects a secondary consequence of chronic inflammatory states rather than a direct pathogenic driver [37]. Given that hypercobalaminaemia has been linked to inflammation-related conditions like autoimmune diseases, further investigation into its mechanisms and reproducibility in future NCP studies could provide valuable insights [38]. Nevertheless, the therapeutic effectiveness of vitamin therapy in the context of NCP may be more pronounced in NCP patients exhibiting vitamin deficiencies. Whilst this study provides preliminary insights into vitamin abnormalities in NCP, we also recognise that the study’s omission of follow-up interventions correcting the vitamin imbalances and longitudinal symptom assessment following treatment limits the interpretation of the evidence.

The role of vitamin B12 in NCP has been explored in an interventional study by Ozen et al., which assessed 90 severe DED patients with NCP, categorising the patients into two cohorts based on their serum vitamin B12 status [39]. Both cohorts received daily artificial tear drops and cyclosporine eye drops, while the vitamin B12-deficient group additionally underwent intramuscular (IM) vitamin B12 therapy consisting of 1000 μg daily for 1 week, followed by monthly injections. Assessment of NCP through OSDI scoring revealed significant amelioration in both treatment arms after 12 weeks, with a more substantial decrease in overall OSDI scores and the individual scores of the pain-specific 3rd OSDI question (‘Have you experienced painful or sore eyes during the last week?’) within the vitamin B12-treated group. However, the difference in mean score reductions between the groups was not statistically significant. This finding was further supported by a case of NCP, which was treated with daily IM vitamin B12 supplementation of 1000 μg for 1 week, followed by monthly administration of 1000 μg over 6 months [40]. Despite demonstrating no response to a 5-month regimen of artificial tears and topical cyclosporine administered daily, the patient was reported to achieve complete symptom resolution within 3 weeks of initiating vitamin B12 supplementation. This highlights the potential role of vitamin B12 in the management of NCP.

Additionally, the role of vitamin D deficiency was also documented in a case of NCP [41]. The patient presented with burning pain, photophobia and foreign body sensation refractory to topical treatment with a history of laser refractive surgery 6 years ago. The case demonstrated a temporal association between vitamin D repletion through 1000 IU daily oral supplementation and symptom improvement, with the patient’s ocular manifestations resolving completely within four days of correcting the identified vitamin D deficiency. The resolution of NCP symptoms following vitamin D repletion reinforces the potential etiological role of hypovitaminosis D in NCP.

### Mechanism of action and potential effects

The pharmacological basis and efficacy of vitamins in NCP therapy remain limited and unclear. Nevertheless, existing studies exploring their effects on other neuropathies and eye-related disorders could yield important clues relevant to their role in managing NCP.

#### Vitamin B3

Vitamin B3 belongs to the water-soluble B complex group and is naturally present in numerous food sources, including bran, yeast, eggs, peanuts, red meat, fish, legumes, and seeds. This molecule is hypothesised to ameliorate NCP through multiple pathways, with one key mechanism being the increase in blood nicotinamide adenine dinucleotide (NAD^+^) levels [42]. Mechanistic studies utilising rat models have demonstrated that daily vitamin B3 supplementation of 200 mg/kg significantly elevated circulating blood NAD^+^ levels by over 50% following a month of treatment [43]. Current understanding suggests that the biosynthesis of NAD^+^ via Vitamin B3 supplementation occurs in a three-step pathway, with nicotinic acid mononucleotide and nicotinic acid adenine dinucleotide as key intermediates [30, 44]. NAD^+^, an essential redox coenzyme, plays an integral role in mitochondrial oxidative phosphorylation, exerting neuroprotective effects by preventing oxidative stress and axonal degeneration induced by mechanical stress or neurotoxic injury [45–47]. NAD^+^ ameliorates mitochondrial dysfunction through its role as a proton acceptor to generate nicotinamide adenine dinucleotide (NADH), which is oxidised in the electron transport chain, contributing to the mitochondrial proton gradient and allowing for the generation of adenosine triphosphate (ATP) via ATP synthase [48]. Additionally, the neuroregenerative effects of NAD^+^ are stimulated through the upregulation of the activity of an enzyme, sirtuin 2, which targets α-tubulin for deacetylation in the dorsal root ganglia and axons – a contributor implicated in neurodegeneration due to its role in microtubule polymerisation [42]. This offers compelling evidence of how the neuroprotective role of vitamin B3 in NCP may be mediated through its antioxidant properties.

Vitamin B3 supplementation has also been thought to mediate NCP through anti-inflammatory mechanisms. In a clinical study, daily oral administration of 1 g nicotinamide riboside in 12 adult males for 21 days significantly downregulated serum inflammatory cytokine levels of interleukin (IL)-6, IL-5 and IL-2, inducing anti-inflammatory signatures [49]. In vitro and in vivo studies have shown that the anti-inflammatory effects of vitamin B3 stem from its agonistic effect on hydroxycarboxylic acid receptor 2 (Hcar2), highly expressed on immune cells, including monocytes and macrophages [50]. Hcar2 agonism on immune cells downregulates nuclear factor-kappa beta (NF-κB) transcriptional signalling with consequent reduction in pro-inflammatory cytokine production [51]. This highlights the possible anti-inflammatory mechanism of vitamin B3 supplementation for the management of NCP. Thus, vitamin B3 may exert therapeutic effects in NCP through three mechanisms: antioxidative effects via NADH production, neuroregenerative processes driven by sirtuin 2 upregulation and anti-inflammatory action via Hcar2 agonism.

#### Vitamin B12

Vitamin B12 is the largest and most complex vitamin in the human body, which serves as a critical cofactor in DNA synthesis and cellular energy production [52]. Vitamin B12 supplementation has been employed to manage pain, particularly for herpes-related neuropathic pain and diabetic neuropathy [53–55]. Vitamin B12’s therapeutic impact on NCP management has been suggested to involve a combination of neuroregenerative, anti-oxidant, and antinociceptive mechanisms. Preclinical studies showed that topical application of 0.05% vitamin B12 eyedrops 4 times daily for 10 days stimulated corneal re-epithelialisation and corneal nerve regeneration in rat models compared to vehicle-treated models through significantly increased neurofilament and β-III tubulin expression [56]. Vitamin B12 has also been shown to improve nerve conduction velocity and subsequent wound healing, alleviating neuropathic pain in diabetic neuropathy patients in several studies [57, 58]. Additionally, Vitamin B12 supplementation suppressed aberrant spontaneous neuronal activity in dorsal root ganglia of rodent models, effectively mitigating associated allodynia and hyperalgesia, demonstrating its anti-allodynic properties [59]. Current preclinical evidence, along with data from other neuropathic disorders, supports the notion that vitamin B12 may contribute to neuroprotection by enhancing nerve regeneration, improving nerve conduction velocity and inhibiting ectopic spontaneous activity in neuropathic pain.

The antioxidative activity of Vitamin B12 has been illustrated in Macri et al*.* [60]. Administration of topical vitamin B12 eye drops four times daily for a month significantly lowered oxidative stress levels in DED patients, assessed via lipid peroxidation assays, relative to vehicle-treated patients [60]. Suppressing oxidative stress may be crucial in managing neuropathic pain, as oxidative stress-induced mitochondrial dysfunction is a global pathogenic factor, contributing to cellular damage, axonal degeneration, and inflammation [61].

Lastly, the antinociceptive effects of vitamin B12 may also account for the improvement of NCP symptoms observed after vitamin B12 supplementation. Studies have shown that the antinociceptive effects of vitamin B12 are achieved through the increased serotonin and noradrenaline levels in different brain regions, enhancing the inhibitory regulation of the nociceptive system [62]. Similarly, its ability to enhance cyclic guanosine monophosphate (cGMP) levels within the nitric oxide-cGMP signalling pathway—a critical mediator of pain modulation—along with its inhibition of the cyclooxygenase pathway and prostaglandin E2 (PGE2), may offer additional mechanistic insights into Vitamin B12’s analgesic properties [63, 64]. In summary, the therapeutic potential of vitamin B12 may primarily be attributed to its neuroregenerative effects, such as the upregulation of neurofilament and β-III tubulin expression, antinociceptive effects via modulation of inhibitory pathways and antioxidative effects.

#### Vitamin D

Vitamin D, a secosteroid, acts as an essential metabolite for various human physiological processes, including calcium homeostasis, immunomodulation, cellular differentiation, and proliferation. Vitamin D’s beneficial effects in NCP are thought to be mediated through its anti-inflammatory, analgesic, neuroregenerative and antioxidative effects. Vitamin D has been established as an endogenous modulator of immune response and is known for its anti-inflammatory effects [65]. Within the ocular tissue, vitamin D exerts regulatory effects on toll-like receptor (TLR)-mediated inflammation, significantly lowering expression of pro-inflammatory cytokines in human corneal epithelial cells following 24 h treatment of 10^−7^ M vitamin D3 [66, 67]. Similarly, comparable results were observed when 5 μL of 10^−6^ M vitamin D was administered topically to dry eye rat models for 10 days, which attenuated corneal inflammation [68]. This effect was mediated by reduced phosphorylated NF-κB inhibitor alpha (IκBα) levels and NF-κB translocation, as evidenced by immunostaining, resulting in significantly decreased expression of downstream pro-inflammatory cytokines IL-6 and IL-8 [68].

Although the precise mechanisms underlying vitamin D's analgesic effects remain unclear, current evidence has shown that hypovitaminosis D has been implicated in the maladaptive hyperinnervation of nociceptors in skeletal muscle tissues [69]. On a molecular level, vitamin D has been shown to downregulate the synthesis of nitric oxide, a retrograde transmitter that stimulates nociceptive hypersensitivity [70]. Additionally, in vitro studies on human lung fibroblasts showed that exposure to 1 μM vitamin D over 24 h modulated the T-cell response and inhibited fibroblast-derived PGE2 synthesis, an important mediator in pain pathways [71]. Other studies have further postulated vitamin D’s role in modulating the synthesis and release of serotonin and dopamine, key neurotransmitters involved in pain processing [72].

**Fig. 2:**
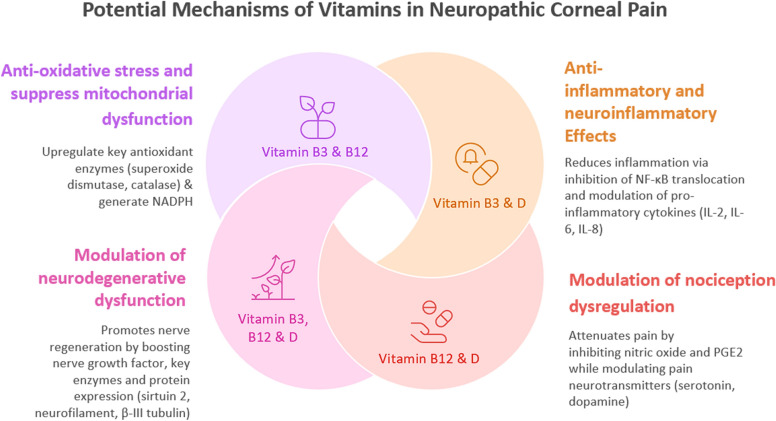
Schematic diagram of the potential mechanisms of vitamins in mitigating neuropathic corneal pain. NADPH, nicotinamide adenine dinucleotide phosphate; NF-κB, nuclear factor kappa B; IL, interleukin; PGE, prostaglandin E

The therapeutic effects of vitamin D may also be attributed to its antioxidative properties, as evidenced by its upregulation of key antioxidant enzymes like superoxide dismutase, catalase, and glutathione peroxidase through the neutralisation of ROS [73]. Furthermore, vitamin D has also demonstrated its neurotrophic capability, stimulating nerve growth factor expression in both peripheral and central dorsal root ganglion neurons innervating the skin and the hippocampus, respectively [74–76]. This suggests its possible neuro-regenerative effects in improving sensory neuron function in NCP. These mechanistic insights of vitamin D’s effects may account for a multimodal mechanism consisting of anti-inflammatory, antioxidative, neuroregenerative, antinociceptive effects for its potential therapeutic effects observed in NCP. Table 1 summarises current evidence regarding vitamin deficiency correlations and the treatment efficacy of specific vitamin subtypes in NCP, while Fig. 2 illustrates the potential pathways by which vitamins exert their effects in NCP.

**Table 1:** Summary of studies investigating the role of vitamins or the efficacy of vitamin supplementation in neuropathic corneal pain

Study	Study design	Vitamin	Key findings	Possible mechanisms of actions
Hamity et al. [34]	Preclinical in vivo rat model study (n = 114)	B3	Paclitaxel-induced corneal hypersensitivity was significantly reversed in rats treated with nicotinamide riboside (500 mg/kg) daily for 28 days compared to placebo-treated rats	• Antioxidative effects via generation of nicotinamide adenine dinucleotide (NADH) • Nerve regeneration: upregulates sirtuin 2 activity • Anti-inflammation: hydroxycarboxylic acid receptor 2 (Hcar2) agonism on immune cells which downregulates inflammatory cytokine production
Bogen et al. [36]	Retrospective cohort study (n = 84)	B2, B6, B12 & D	Vitamins B2 and D deficiencies observed in 31% and 16% of the neuropathic corneal pain (NCP) cohort. Elevated vitamins B6 and B12 observed in 28.9% and 15% of the NCP cohort	Not discussed
Ozen et al. [39]	Prospective interventional cohort study (n = 90)	B12	While conventional treatment (artificial tears/cyclosporine) significantly alleviated NCP symptoms in both B12-deficient and normal groups, adjunct intramuscular (IM) B12 supplementation (1000 μg daily for 1 week, then monthly for 3 months) in B12-deficient group yielded a larger yet statistically insignificant Ocular Surface Disease Index (OSDI) score reduction compared to normal NCP group	• Neuroregenerative: upregulates neurofilament and β-III tubulin expression; improves nerve conduction velocity; inhibits spontaneous ectopic activity
Shetty et al. [40]	Case report	B12	Daily vitamin B12 supplementation (1000 μg for a week followed by a monthly administration of 1000 μg) to a vitamin B12 deficient patient with NCP refractory to dry eye disease (DED) treatment achieved symptom resolution without 3 weeks of treatment initiation	• Antinociceptive: enhances inhibitory regulation of descending inhibitory nociceptive system & inhibition of cyclooxygenase pathway and prostaglandin E2 (PGE2) • Antioxidative effects: suppresses oxidative stress levels
Singman et al. [41]	Case report	D	Correction of vitamin D deficiency (1,000 IU/day for 4 days) in an NCP case refractory to DED treatment achieved symptom resolution within 4 days of treatment initiation	• Anti-inflammation: regulates toll-like receptor (TLR)-mediation inflammation, reduces pro-inflammatory cytokine expression • Antioxidative: upregulates anti-oxidant enzymes (e.g. superoxide dismutase, catalase) • Nerve-regenerative: promotes nerve growth factor • Analgesic: inhibits nitric oxide; inhibits PGE2 production; modulates pain neurotransmitters (e.g. serotonin, dopamine)

## Discussion

The global prevalence of vitamins B and D deficiencies is estimated at approximately 6% and 15.7%, respectively [77, 78]. The underlying reasons why only a small subset of patients develop NCP remain unclear. We hypothesise that this phenomenon may arise from a complex interplay of biochemical and neurobiological susceptibility factors. The coexistence of vitamin deficiencies and predisposing factors for NCP, such as psychological conditions or chronic pain syndrome, may amplify the perception of corneal pain more intensely [79]. Additionally, the coexistence with preexisting neuropathies such as diabetes may also contribute to a reduced threshold for experiencing NCP. The current understanding of NCP in vitamin-deficient populations is limited, indicating the clear need for more in-depth research in this domain.

Current evidence supporting the use of vitamin supplementation in NCP is promising, drawing from both preclinical and clinical data related to the treatment of NCP and broader neuropathic pain syndromes. Additionally, the proposed mechanisms of action associated with vitamin supplementation appear to mitigate key pathological processes driving NCP, thereby lending further support to its therapeutic relevance. Nevertheless, the current body of evidence remains limited; existing studies are limited to preclinical studies with a small volume of observational clinical studies. There are currently no clinical trials specifically examining the role of vitamin supplementation in NCP. This highlights a current reliance on extrapolated data from animal models or other neuropathic pain states, with a paucity of data from interventional studies specifically targeting NCP. Furthermore, there is a heterogeneity in vitamin formulations and doses across these studies.

### Future directions

Current studies offer an encouraging narrative to support the potential association between vitamin abnormalities and NCP pathogenesis, while supporting vitamin supplementation as a viable treatment approach for NCP. Nevertheless, research investigating the role of vitamin-related abnormalities in NCP remains limited and additional investigations are essential to validate its role before adoption in clinical contexts. Although existing literature has partially delineated the mechanistic pathways of vitamin supplementation in neuropathic conditions, current research on these mechanisms remains largely limited to other conditions. This includes the current understanding of vitamin pharmacokinetics and pharmacodynamics within the cornea, which remains poorly characterized due to the early and evolving nature of research in this field. The pharmacokinetics and pharmacodynamics in corneal tissues are closely tied to the dosing and routes of administration, both of which currently lack standardisation. Whilst the efficacy of vitamin supplementation has been well-established in peripheral neuropathies [80, 81], direct pharmacokinetic assessments of vitamin levels in peripheral nerve tissues remain lacking. Further studies are warranted to advance our knowledge of the disease pathology and to evaluate the potential interactions between nutritional interventions and the condition, with particular emphasis on the pharmacodynamics of vitamins and their specific target tissues in NCP.

Next, the role of vitamin supplementation should be tested in large-scale multicenter clinical studies and adequately powered clinical trials to establish conclusive evidence regarding the role of vitamins in NCP management. The current body of research on vitamin supplementation in NCP consists principally of observational data and in vivo animal studies. The role of serum vitamin levels as a biomarker of NCP severity or therapeutic response warrants future well-designed studies, as their interpretation may be confounded by other comorbidities such as chronic dry eye and diabetic corneal neuropathy, inherent to the multifactorial nature of NCP. Additionally, comparing combination versus monotherapy in future clinical trials will enhance our understanding of vitamin supplementation in NCP. Other avenues for research may include dietary lifestyle interventions, including low-fat plant-based, caloric-restricted, and potassium-reduced diets, which have been associated with neuroprotective and anti-inflammatory benefits in neuropathic pain and may hold promise for the prevention and treatment of NCP [82]. Continued and rigorous clinical assessments of the efficacy of vitamin supplementation along with the optimisation of treatment parameters such as dosing regimens, treatment duration and administration methods, are crucial for validating real-world therapeutic effects for NCP. Topical vitamin formulations may offer advantages in achieving higher vitamin concentrations in the targeted tissue (cornea) compared to IM and oral routes [83]. Furthermore, topical administration may mitigate the risk of dose-dependent systemic adverse effects associated with oral and IM administrations. Nevertheless, additional studies are needed to substantiate the pharmacokinetic advantages of different vitamin supplementation delivery pathways.

## Conclusion

This review provides an overview of the current therapeutic landscape for NCP, the potential implication of vitamin dysregulation and the clinical effectiveness of vitamin supplementation in the context of NCP. Current studies have demonstrated the viability of vitamins B3, B12 and D supplementation as a therapeutic option in managing NCP. Beyond their nutritional role, the various vitamins have been proposed to mitigate NCP pathology through their anti-inflammatory effects, nerve regeneration and regulation of nociceptive effects. Given the established safety profile and commercial accessibility of vitamin supplements, they represent a promising candidate for integration into NCP treatment algorithms to potentially augment existing therapeutic approaches. Nonetheless, vitamin supplementation should be represented in larger, well-powered studies to further establish its therapeutic relevance in NCP. While substantial progress remains to be made, we propose that sustained investigative efforts will deepen our understanding of the condition and provide further validation for emerging therapeutic strategies, including vitamin supplementation.

## Data Availability

Not applicable.

## Abbreviations

NCP: Neuropathic corneal pain
DED: Dry eye disease
IVCM: In vivo confocal microscopy
OPAS: Ocular Pain Assessment Survey
OSDI: Ocular Surface Disease Index
SNRI: Serotonin-norepinephrine reuptake inhibitor
NAD^+^: Nicotinamide adenine dinucleotide
BMP3: Bone morphogenetic protein 3
DPYL2: Dihydropyrimidinase-related protein 2
IL: Interleukin
Hcar2: Hydroxycarboxylic acid receptor 2
NF-κB: Nuclear factor-kappa beta
cGMP: Cyclic guanosine monophosphate
PGE2: Prostaglandin E2
TLR: Toll-like receptor
